# A proposed minimum skill set for university graduates to meet the informatics needs and challenges of the "-omics" era

**DOI:** 10.1186/1471-2164-10-S3-S36

**Published:** 2009-12-03

**Authors:** Tin Wee Tan, Shen Jean Lim, Asif M Khan, Shoba Ranganathan

**Affiliations:** 1 Department of Biochemistry, Yong Loo Lin School of Medicine, National University of Singapore, 8 Medical Drive, Singapore 117597; 2 Department of Chemistry and Biomolecular Sciences and ARC Centre of Excellence in Bioinformatics, Macquarie University, NSW 2109, Australia

## Abstract

**Background:**

The development of high throughput experimental technologies have given rise to the "-omics" era where terabyte-scale datasets for systems-level measurements of various cellular and molecular phenomena pose considerable challenges in data processing and extraction of biological meaning. Moreover, it has created an unmet need for the effective integration of these datasets to achieve insights into biological systems. While it has increased the demand for bioinformatics experts who can interface with biologists, it has also raised the requirement for biologists to possess a basic capability in bioinformatics and to communicate seamlessly with these experts. This may be achieved by embedding in their undergraduate and graduate life science education, basic training in bioinformatics geared towards acquiring a minimum skill set in computation and informatics.

**Results:**

Based on previous attempts to define curricula suitable for addressing the bioinformatics capability gap, an initiative was taken during the Workshops on Education in Bioinformatics and Computational Biology (WEBCB) in 2008 and 2009 to identify a minimum skill set for the training of future bioinformaticians and molecular biologists with informatics capabilities. The minimum skill set proposed is cross-disciplinary in nature, involving a combination of knowledge and proficiency from the fields of biology, computer science, mathematics and statistics, and can be tailored to the needs of the "-omics".

**Conclusion:**

The proposed bioinformatics minimum skill set serves as a guideline for biology curriculum design and development in universities at both the undergraduate and graduate levels.

## Background

The advancement of modern technology in recent years has given rise to the development of innovative high throughput, automated biotechnologies such as next-generation and whole-genome sequencing technologies that allows for the generation of genome-scale data providing unprecedented systems-level measurements for detecting temporal and conditional changes in various types of cellular components [1]. The generation of such data has expanded and transformed the practice of traditional biological research, signaling the dawn of the "-omics" era [2].

The "-omics" era has brought with it numerous challenges and hurdles, the most significant being the processing of the massive amount of datasets and the extraction of biological meaning from them [3]. Another considerable challenge lies in the effective integration of these datasets to achieve global insights into cellular and molecular behavior and biological systems [3,4]. In view of these challenges, bioinformatics has proved to be increasingly vital for the "-omics" to meet the critical need for the management, analysis and integration of the massive-scale datasets [5]. This has increased the demand for highly trained and experienced bioinformatics experts who can handle the data deluge as well as interface with biologists. The number of courses in bioinformatics and computational biology producing such specialists has indeed burgeoned.

Concurrently, it has also raised the requirement for biologists to possess an ever-increasing capability in bioinformatics and to communicate seamlessly with the bioinformatics specialists and experts. This requirement may be achieved by various means, including ad hoc training courses, bridging Masters programs, as well as embedding in the typical undergraduate and graduate life science education, basic training in bioinformatics geared towards acquiring a minimum skill set in computation and informatics.

Various attempts have been made to address and define the current needs and issues involved in bioinformatics education. In 2001, the Workshop on Education in Bioinformatics (WEB) http://surya.bic.nus.edu.sg/web01/ was launched at the 2001 International Conference on Intelligent Systems for Molecular Biology (ISMB) as a satellite meeting to provide, for the first time, a platform for bioinformatics educators to discuss fundamental educational and pedagogical issues determining the nature, extent, and content of, and delivery tools available for, bioinformatics degree and training programs, and to provide focus points and suggestions for improvement of nascent degree programs [6]. More recently in 2009, the RECOMB Bioinformatics Education Conference http://casb.ucsd.edu/bioed/ has focused its attention not just on equipping biology students with new skills, but also on shifting the mindset towards more computational courses in standard biology curricula, even though the best way to achieve this remains elusive [7]. Specific to an Asian context, the 3rd East Asia Bioinformation Network meeting http://eabn.apbionet.org/3eabn08/docs.shtml, held in Singapore (April 2008) witnessed the proposal for minimum skills required of biologists in bioinformatics and biocomputation (msrBIC), particularly for resource-lacking developing countries of the Association for Southeast Asian Nations (ASEAN).

The first Workshop on Education in Bioinformatics and Computational Biology (WEBCB) was conducted in October 2008 with the main purpose of identifying a minimum skill set for new generation biologists to cope with the main informatics needs and challenges of the "-omics" era to be used as a guideline for the development and design of a new bioinformatics or integrated life science curriculum in the future. As follow-up, a second WEBCB panel discussion session was held on September 8, 2009, to present the 2008 report and collate the conclusions from the two WEBCB meetings.

## Results and discussion

### Proposed minimum skill set as deliverables of bioinformatics education

The 2008 one-day workshop, jointly organized by (APBioNet), International Union of Biochemistry and Molecular Biology (IUBMB) and The Federation of Asian and Oceanian Biochemists and Molecular Biologists (FAOBMB) was attended by 56 students and bioinformatics researchers and educationists across various countries; the second 2009 follow-up workshop was also well attended. While all participants recognized bioinformatics as an inter-disciplinary field encompassing concepts and techniques from the areas of biology, computer science, as well as mathematics and statistics, as expected, not all could agree on a definitive set of topics for implementation in a standard curriculum.

Some of the major challenges in bioinformatics education raised included the need for unifying the diverse disciplines within the field of bioinformatics, imparting to students thinking skills to handle bioinformatics problems and establishing universal standards for bioinformatics courses at different levels. Bioinformatics experts originating from computer science tended to request for a higher content of the "hard" sciences, pushing for more mathematical and statistical content. Those with biology backgrounds, generally not formally trained in computing, would steer towards a more conservative, tool-user approach to bioinformatics which the computing community deems as inadequate. The lack of consensus is reflected in the absence of any clear definitive standard yardstick one can apply to graduates of a standard university biology course to measure their competence in handling the computational challenges of today's research environment.

The outcome of these and other workshops thus gravitated towards the definition of a standard set of minimum skills for competency in bioinformatics research that might be agreed upon by all participants. The minimum skill set, as described next, may then be tested against any existing curriculum for comparison or benchmarking.

### Definition of minimum skill set

An undergraduate life science curriculum should endeavour to imbue in their students with the necessary skill sets to survive and thrive in the "-omics" age of research, where there is an over-abundance of data, information and knowledge and a scarcity of ability to transform that into insight, knowledge, positive returns and outcomes to the society that has subsidized the tertiary educational process.

Whereas different institutions have differing emphasis and criteria for a successful modern life science curriculum, there are signs of some consensus in defining a minimum skill set for bioinformatics. Instead of tackling questions such as "How to teach bioinformatics" or "What to teach in bioinformatics to biology students", which baffle many educationists as they cope with competing demands on typically packed biology schedules, defining a minimum skill set approaches the problem from the product of the curriculum, the trained student. What are the skills one would reasonably expect from a graduate of a biology course? What knowledge, skills and abilities should they possess? What can we expect them to do proficiently?

### Minimum bioinformatics skill Set

The minimum bioinformatics skill set expected of a biology graduate, as listed below, represents a distillation of the collective thoughts and ideas of multiple researchers in several contexts of developed countries which may not be applicable to the needs or capabilities of resource-limited developing countries whose societies have not yet entered the knowledge-based economy. The minimum skill sets for such developed countries should be more than that for developing countries, for example, such as that enunciated for countries in the Association for South-east Asian Nations (ASEAN) during the 3rd East Asia Bioinformation Network (3rd EABN) 2008, Singapore held in April 2008 http://eabn.apbionet.org/3eabn08/docs.shtml

1. Basic essential knowledge in the specific domains of computer science, statistics and mathematics that intersect with modern biology

Modern biology is increasingly open to application of specific domains of computer science, statistics and mathematics. From techniques of programming such as dynamic programming underlying the basis of sequence comparison, alignment and analysis, to knowledge representation, machine learning and data mining in recent years, covering artificial neural networks (ANN), genetic algorithms, hidden Markov models (HMM), support vector machine (SVM), these have had tremendous impact in modern biology. Traditional topics in statistics and probability, integral and differential calculus, linear algebra, and so on, must not be forgotten either. Such topics are already present in today's curriculum of diverse disciplines from financial engineering and economics to physics and chemistry, and so biology should be no less quantitatively rigorous. The main contention here seems to be how such mathematical and computational ideas should be conveyed to undergraduate biologists and the associated pedagogy involved in a packed curriculum. Can we develop a meaningful understanding of such ideas without knowledge of programming or some degree of mathematical formalism?

2. Expertise in communicating and representing biological knowledge and processes in mathematical, statistical and computing terms and concepts

Much of bioinformatics today may be taught as a course in how to use bioinformatics tools and indeed, many textbooks are starting to look like cookbooks of recipes and protocols which biology students can follow without understanding the underlying principles. They often end up misusing these techniques and misinterpreting the results. Based on a solid foundation on the concepts, and a lack of fear in dealing with mathematical, statistical and computing ideas, biologists still need the ability to represent biological knowledge in such terms and to communicate with people from other disciplines in their language. Such cross-disciplinary communications are vital in today's integrative research environment where a physicist may be locking intellectual horns with clinicians, or a bioengineer with an ethicist. A curriculum that includes training students in communicating scientific ideas and presenting knowledge can be transformative. In representing and abstracting biological knowledge in mathematical, statistical or computing terms, we take it to another level of rigour needed in today's world of the "-omics".

3. Ability to use and/or develop efficient bioinformatics and biocomputational tools and techniques for the acquisition, interpretation, analysis, prediction, modeling, simulation and visualization of experimental and other biological data

The range and breadth of the tools and techniques available today almost defy cataloguing. The average undergraduate curriculum is bound to include some explanation of the Basic Local Alignment Search Tool (BLAST), multiple sequence alignment or the drawing and interpretation of some phylogenetic tree. The more advanced courses may even go into SVM predictions or graphical visualization of 3D models of structure, homology modeling or ligand-receptor docking. Any card-carrying biologist must have some ability to use and in some cases, modify and develop these tools to fit the task. Indeed, one must be able to traverse a bioinformatics software application from its basic usage to a critical understanding of the principles underlying the application, including comprehension of documentation and publication describing the application.

Moreover, these tasks increasingly form an interconnected pipeline of workflows in today's research laboratory. Picking up a few scripting (bash, Perl or Python) and programming languages (Java or C) and mastering at least one, may not be too far fetched an idea to include in more ambitious curricula.

4. Proficiency in the search, retrieval, processing, curation, organization, classification, management, and dissemination of biological data and information in databases for deriving biological insight and knowledge discovery

The earliest activities of bioinformatics included the building of databases. Knowledge of the structure and content of basic primary databases such as those from NCBI down to boutique ones is essential as much as the ability to manipulate and process large databases. As more data is produced by the "-omics", and now with next generation sequencing in the fore, another wave of data deluge is to be expected. In making sense of the voluminous data generated in today's high throughput laboratory research, topics in database design and curation and the organization of the information through use of controlled vocabularies, ontologies and other aspects of information science is inevitable in the undergraduate curriculum.

Many of our graduates end up in such research laboratories and equipping them with basic proficiency in dealing with database transformation or extraction of data subsets is an essential skill. Younger biologists more in tune with the Internet age are often expected to build and run their laboratory website and maintain databases of data created in their laboratory. Information dissemination over the electronic network is a basic routine task. In fact, every biological research endeavour today starts with the search and retrieval of information from a simple Google query to a complex interrogation of specialty databases. Scientific literature is almost entirely electronic today, and bibliographic reference management to bibliometric analysis of the principal investigator's H-index is something many take for granted. Therefore, proficiency in all these tasks leads to a better competitive chance at deriving biological insight and knowledge discovery. Mastering SQL in this context or learning how to apply PHP to connect a web form with a database query would be helpful, perhaps as much as understanding the underlying principles of network connectivity and the TCP/IP Internet protocol.

In this way, a well-trained biologist should be able to reduce a biological problem to its informatic sources, and to analyse the data computationally and algorithmically, and wherever possible, to code simple programs to facilitate the integrative process of solving complex biological problems.

5. Critical thinking and problem solving skills in quantitative aspects of biology

As in all basic sciences at the undergraduate level and beyond, critical thinking is an essential component of university education. Coupled with problem solving, the combination is formidable, particularly when applied to the challenge of shifting biology education from a totally qualitative exercise to a balance of the quantitative with the qualitative. Any biology student graduating with strength in critical thought and ability in problem solving would be sought after for employment, whether in biological research or otherwise.

The minimum skill set of five key areas proposed above is cross-disciplinary in nature, involving a combination of knowledge and proficiency from the fields of biology, computer science, mathematics and statistics. It is flexible enough to be curriculum-tailored to the needs of the "-omics". It serves as a guideline for bioinformatics curriculum design in universities at both the undergraduate and graduate levels. Such curricula equip students with knowledge in particular biological domains and software applications and provide bioinformatics training and education that nurture transferrable skills such as critical thinking and problem-solving.

Based on a curriculum covering the minimum skill set proposed, an undergraduate life science student, in addition to being well-versed with biological research techniques, should gain exposure to most components in the skill set, accompanied with a limited small-scale application and integration of a few selected bioinformatics tools and techniques to a research project. A student studying bioinformatics at the Master's level, on the other hand, should minimally possess knowledge on all components in the minimum skill set, in addition to in-depth applications of a variety of bioinformatics tools and techniques to a biological problem, while one at the PhD level should gain proficiency in all components described in the minimum skill set, both in theory and application to a wide enough body of problems covered in the PhD thesis.

### Benchmarking the minimum skill set against published bioinformatics curricula

Following the proposal of a minimum skill set, its usefulness in evaluating the quality of bioinformatics education was assessed by comparison against several university bioinformatics curricula described in the literature.

In 2008, Koch *et al*. reported a basic bioinformatics curriculum, consisting of topics and skills taught in nearly all universities and research institutes in Germany [8]. In agreement with the proposed minimum skill set, the authors recognized bioinformatics as a strongly multi-disciplinary field where fundamentals in biology, mathematics and computer science are critical. In general, the basic bioinformatics curriculum they described, at both the undergraduate and master's level, fit the scope of the proposed minimum skill set, imparting students with essential knowledge and principles in the relevant fields of biology, mathematics and computer science. A prototypical curriculum example provided is the consecutive bioinformatics bachelor's and master's program implemented in The Free University Berlin. The program covers the fundamentals in the areas of mathematics, computer science, biochemistry/chemistry and physiology, which represent important elements in the proposed skill set.

On the other hand, Yale University has developed an inter-departmental Ph.D. program in computational biology and bioinformatics which focuses on three broad core areas of competency, which include computational biology and bioinformatics, biological sciences and informatics (including computer science, statistics, and applied mathematics) [9]. The areas of minimum expected competency listed, namely in biology - introductory biology and biochemistry, computer science - introduction to programming, concepts, techniques, and applications of computer science and data structures (arrays, stacks, queues, lists, trees, heaps, and graphs), sorting and searching, storage allocation and management, and data abstraction, mathematics and statistics - multivariate calculus, linear algebra, and introductory statistics, are found to fall within the typical scope, and even surpassing the requirements (in the area of statistics) of the proposed minimum skill set.

In the allied field of biomedical informatics, Altman and Klein at Stanford [10] have built an integrated PhD, MS, certificate program and undergraduate major in biomedical computation. Key skills covered in their training match the skill set described here. Finally, Wingreen and Botstein [11] in Princeton University describe a first year graduate course in quantitative biology using a close reading of classic and pedagogically most successful papers whose nature and quality function as "vehicles for teaching both biology and quantitative analysis". Here the basic skill set, though advanced in nature, are fundamentally similar. Physics graduates share their strengths in quantitative science and biology graduates share their knowledge of biology as they tackle biological problems exemplified by the papers in a quantitative, informational and computational manner.

## Conclusion

The minimum skill set in bioinformatics recommended for the future generations of biological/life scientists serves as a guideline for the development of an integrated life sciences curriculum across both the undergraduate and graduate levels. This should also encompasses both lifelong and transferrable skills such as critical thinking and problem-solving skills, in addition to the essential knowledge in the specific domains of biology, computer science, statistics and mathematics, which will prove useful in meeting the informatics needs and challenges of the "-omics" era in various ways. Most significantly, the acquired expertise in biological and mathematical knowledge and computer languages will facilitate mining of high throughput data and information to extract valuable biological meaning, and the development of efficient bioinformatics tools for the analysis, prediction and modeling of these experimental data. At the same time, proficiency in database management and Internet technologies will allow for the creation and maintenance of online websites and databases for the storage, management, presentation and sharing of high throughput experimental data, to further boost research growth and progress in the field.

A sampling of bioinformatics curricula reported in developed countries appear to fulfill and surpass the minimum skill set requirements. For developing countries, which are still lagging in the practice of high throughput biological research, these skill set requirements would more than enable the best of their graduates to be equipped with a fundamental base to build on should they intend to venture overseas for graduate studies. As the field of bioinformatics rapidly progresses, the expectation of biology graduates will increase, and this minimum skill set is expected to change and expand in scope, particularly as systems biology starts to mature. The question still remains, how an undergraduate or graduate life science curriculum already packed with a full schedule of the fundamentals to the latest developments in biology can cope with the additional bioinformatics demands of the new biology.

## Methods

### Workshop on Education in Bioinformatics and Computational Biology (WEBCB)

The first Workshop on Education in Bioinformatics and Computational Biology (WEBCB) was held on October 23, 2008 in the National Yang Ming University, Taipei, as part of the seventh International Conference in Bioinformatics (InCoB) held in the same venue. A total of three distinguished panelists across different research and education institutions were invited for the panel discussion workshop.

The second follow-up workshop was held on September 7, 2009 in Matrix, Biopolis, Singapore, as part of the International Conference in Bioinformatics (InCoB) with three invited panelists.

## Competing interests

The authors declare that they have no competing interests.

## Authors' contributions

TTW proposed the study, while all authors performed the relevant research and contributed to the manuscript. All authors have read and approved of this manuscript.
